# Li_3_TiCl_6_ as ionic conductive and compressible positive electrode active material for all-solid-state lithium-based batteries

**DOI:** 10.1038/s41467-023-37122-7

**Published:** 2023-03-13

**Authors:** Kai Wang, Zhenqi Gu, Zhiwei Xi, Lv Hu, Cheng Ma

**Affiliations:** 1 Hefei National Research Center for Physical Sciences at the Microscale, CAS Key Laboratory of Materials for Energy Conversion, Department of Materials Science and Engineering, University of Science and Technology of China, Hefei, Anhui 230026 China; 2 School of Materials & Energy, Lanzhou University, Lanzhou, Gansu 730000 China; 3 National Synchrotron Radiation Laboratory, Hefei, Anhui 230026 China

**Keywords:** Batteries, Materials chemistry, Materials for energy and catalysis, Energy storage, Solid-phase synthesis

## Abstract

The development of energy-dense all-solid-state Li-based batteries requires positive electrode active materials that are ionic conductive and compressible at room temperature. Indeed, these material properties could contribute to a sensible reduction of the amount of the solid-state electrolyte in the composite electrode, thus, enabling higher mass loading of active materials. Here, we propose the synthesis and use of lithium titanium chloride (Li_3_TiCl_6_) as room-temperature ionic conductive (i.e., 1.04 mS cm^−1^ at 25 °C) and compressible active materials for all-solid-state Li-based batteries. When a composite positive electrode comprising 95 wt.% of Li_3_TiCl_6_ is tested in combination with a Li-In alloy negative electrode and Li_6_PS_5_Cl/Li_2_ZrCl_6_ solid-state electrolytes, an initial discharge capacity of about 90 mAh g^−1^ and an average cell discharge voltage of about 2.53 V are obtained. Furthermore, a capacity retention of more than 62% is attainable after 2500 cycles at 92.5 mA g^−1^ and 25 °C with an applied external pressure of 1.5 tons. We also report the assembly and testing of a “single Li_3_TiCl_6_” cell where this chloride material is used as the solid electrolyte, negative electrode and positive electrode.

## Abstract (web summary)

An ideal positive electrode for all-solid-state Li batteries should be ionic conductive and compressible. However, this is not possible with state-of-the-art metal oxides. Here, the authors demonstrate the use of an ionic conductive metal chloride as compressible positive electrode active material.

## Introduction

The overall performance of a Li-ion battery is limited by the positive electrode active material^1–6^. Over the past few decades, the most used positive electrode active materials were oxides, such as LiCoO_2_, LiNi_1-x-y_Mn_x_Co_y_O_2_, LiFePO_4_, and LiNi_0.5_Mn_1.5_O_4_^1–6^. However, with the increasing urgent demand of replacing flammable liquid non-aqueous electrolyte solutions with safer solid-state inorganic electrolytes, this situation might change: the desired requirements for positive electrodes in all-solid-state Li batteries (ASSLBs) are not the same as those in commercial Li-ion batteries using liquid electrolyte.

The difference lies in at least two aspects. First of all, with the liquid electrolyte replaced by solid ones, the ionic conductivity of positive electrodes would become more important to the cell performance^2,5^. In order to realize efficient ion transport in the positive electrode, the composite active material also contains a certain volume fraction of solid-state electrolyte^7,8^. Since most solid electrolytes exhibit higher density than their liquid counterparts (2−5 *vs.* 1.28 g cm^−3^ at 25 °C)^9,10^, filling the same volume fraction of a given composite positive electrode using the former would lead to a higher mass fraction of the electrolyte, and consequently a lower mass loading of the active material. Nevertheless, if the positive electrode can enable efficient ion transport, the composite positive electrode would no longer need much (or any) solid electrolyte incorporated^11^. The resulting improvement in energy density could be significant. According to Sun et al.’s estimation on a Li | Li_6_PS_5_Cl | LiNi_0.8_Mn_0.1_Co_0.1_O_2_ battery^12^, the decrease of solid electrolyte content from 30 to 10 wt% in the composite positive electrode would increase the specific energy from 320 Wh kg^–1^ to about 426 Wh kg^–1^. Secondly, unlike the situation for Li-ion batteries using carbonate-based fluorinated non-aqueous liquid electrolyte solutions, compressibility is in fact highly desired for the positive electrode in ASSLBs^11,13^. During prolonged cycling, the emergence of cracks in particles of the active material is inevitable^14–20^. Since solid electrolytes cannot spontaneously fill the developed cracks like liquid^14,17,19,20^, the crack occurring in the brittle, non-compressible positive electrode particles would actually break the pathways for Li-ions to migrate across^15,18^. However, should the positive electrode be compressible, its particle morphology would adapt to this change under external pressure and mitigate the situation^21,22^. Clearly, the two characteristics mentioned above, i.e., high ionic conductivity and good compressibility, cannot be easily realized in oxides; in particular, brittleness is the common feature for nearly all the oxides^23^. Therefore, it seems reasonable to consider non-oxide compounds instead. Nevertheless, related efforts are rare^13,24^. Only a few sulfides that had been studied as positive electrodes before, e.g., Li_6_PS_5_Cl^25^, Li_10_GeP_2_S_12_^26^, are promising in both aspects, but their voltages are far below the level of commercial positive electrodes such as LiCoO_2_ and LiFePO_4_^1,2,5,6^. The positive electrode material that simultaneously possesses high ionic conductivity, excellent compressibility, and a decent voltage has not been identified.

Here, we report Li_3_TiCl_6_ as positive electrode active material. With a discharge voltage close to that of LiFePO_4_, it shows a high ionic conductivity of 1.04 mS cm^–1^ at 25 °C, and is easily compressible like most chlorides. As a result, the fraction of Li_3_TiCl_6_ in the composite positive electrode can reach 95 wt% (the other 5 wt% is carbon black), which surpasses those for the ASSLBs in literature (typically below 80 wt%^27–32^), but still delivers good battery performances. When the all-solid-state cell assembled using such a composite positive electrode was charged and discharged under 95.2 mA g^−1^ at 25 °C, the capacity retention was above 80% for 388 cycles; even after 2500 cycles, a capacity retention of 62.3% was still sustained. These results point out that the barely explored transition-metal chlorides may possess desirable characteristics for the positive electrodes in ASSLBs, and are thus worthy of more extensive, in-depth exploration in future research.

## Results and Discussion

**Fig. 1:**
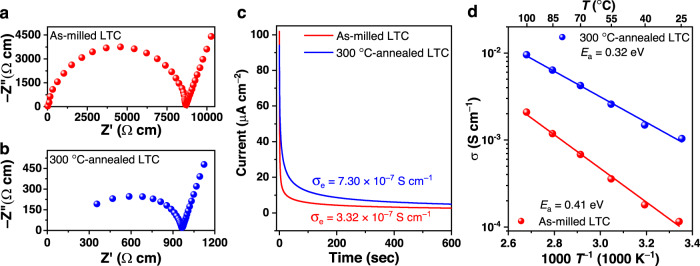
Conductivity of LTC with different processing conditions. **a**, **b** Nyquist plots of the as-milled (**a**) and 300 °C-annealed LTC materials (**b**) at 25 °C. **c** The transient current behavior under an applied 1.0 V DC bias on the as-milled and 300 °C-annealed LTC pellets with stainless-steel electrodes. **d** Arrhenius plots of the as-milled and 300 °C-annealed LTC materials.

Li_3_TiCl_6_ (LTC) was mechanochemically synthesized from a stoichiometric mixture of LiCl and TiCl_3_. As indicated in Fig. 1a, the as-milled LTC showed an ionic conductivity of 1.15 × 10^–4^ S cm^–1^ at 25 °C. After annealing at 300 °C, its ionic conductivity reaches 1.04 × 10^–3^ S cm^–1^ at 25 °C (Fig. 1b). The electronic conductivities determined using the Hebb–Wagner polarization method were 3.32 × 10^–7^ S cm^–1^ and 7.30 × 10^–7^ S cm^–1^ for the as-milled and 300 °C-annealed materials, respectively (Fig. 1c). Consistent with the ionic conductivities at 25 °C, the activation energy of the 300 °C-annealed LTC (0.32 eV) was lower than that of the as-milled LTC (0.41 eV), as indicated in Fig. 1d. According to these results, one of the two desired characteristics raised in Introduction has been successfully realized in the 300 °C-annealed LTC material: its ionic conductivities at 25 °C is above 1 mS cm^−1^.

**Fig. 2:**
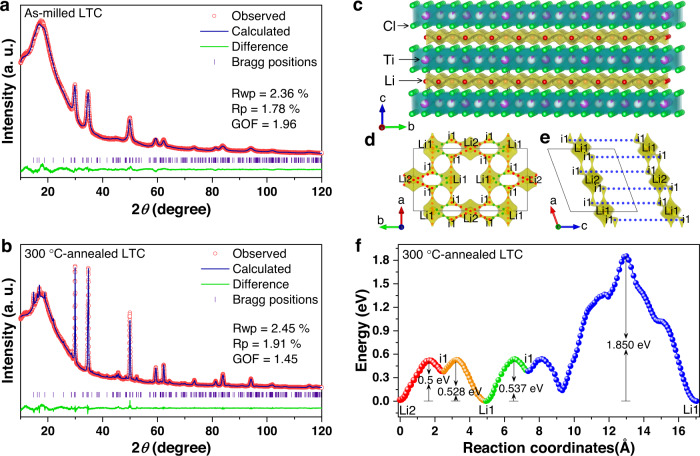
Crystal structure and Li-ion migration behavior of LTC. **a**, **b** Refined XRD patterns of the as-milled (**a**) and 300 °C-annealed LTC (**b**). The broad hump below 30° arises from the Kapton films used to prevent air exposure during measurement. **c** The structural model of LTC superimposed with the Li-ion potential map. Both the structure and BVSE calculation results are from the 300 °C-annealed LTC. **d**−**f** Li-ion migration pathways (**d**, **e**) and the associated energy profiles (**f**). Each energy profile in **f** is plotted in the same color as its corresponding pathway in **d** and **e**.

To determine the crystal structure of LTC, X-ray diffraction (XRD) measurements and Rietveld refinement^33,34^ were performed. Due to the low crystallinity resulting from the intense ball milling, the as-milled LTC material exhibited weaker and more diffuse reflections than the 300 °C-annealed one. Beyond such a distinction, no essential difference was observed between their XRD patterns, suggesting that the overall crystal structures are the same. The LTC materials seem isostructural with the Li_3_InCl_6_ showing the *C2/m* space group^29^; when this structure was used as the initial model (with In replaced with Ti) to refine the XRD patterns of the as-milled and 300 °C-annealed LTC materials, both of them yielded excellent agreement between the experimental and calculated patterns (Fig. 2a, b). The detailed refinement results are summarized in Supplementary Tables 1 and 2, while the corresponding crystal structure is schematically illustrated in Fig. 2c. In order to investigate the ion transport behavior within such an atomic framework, the bond valence site energy (BVSE) method^35,36^ was used to calculate the Li-ion migration pathways and the associated energy barriers; the refined structure of the 300 °C-annealed LTC material (Supplementary Table 2) with improved crystallinity and higher conductivity was used for such a purpose. The result suggests that the ion transport within its a−b planes exhibits a lower effective migration barrier than that across these planes (Fig. 2d−f). This is similar to standard layered oxide positive electrode active materials LiCoO_2_ and LiNi_0.8_Mn_0.1_Co_0.1_O_2_^1–6^. Nevertheless, unlike these oxides, LTC contains lots of vacancies in the transition-metal layer; as shown in Supplementary Table 2, the occupancy of the two Ti sites in LTC are 0.754 and 0.123, respectively. In BVSE calculation, the potentials at both the occupied and empty Ti sites are overly simplified as their average value^35,36^. Under such an assumption, the barrier for Li-ion migration between the a−b planes (1.850 eV, as shown in Fig. 2e, f) would appear too high to allow for any efficient ion transport. However, in reality only the occupied Ti sites would impede ion transport, and the empty ones may still allow Li ions to migrate through. Therefore, the actual ion transport may happen both within and between the a−b planes. In this way, the layered structure of LTC seems to have more Li-ion migration directions available than the layered oxides like LiCoO_2_, whose transition-metal sites are usually fully occupied and largely limit the ion transport within two-dimensional planes^2,6^.

Beyond the appealing ion transport behavior, LTC was also found to be a compressible material. According to the scanning electron microscopy (SEM) observation, the LTC pellet fabricated by cold-pressing under 350 MPa (without any heat treatment) shows a low porosity, as shown in Supplementary Fig. 1a. Consistent with these SEM images, the relative density of the LTC pellet was measured to be 86.1%, larger than those of the pellets made from other compressible solid electrolytes under the same or higher pressures (Supplementary Fig. 1b), including Li_3_YCl_6_ (76−79% under 350 MPa)^37^, Na_2_ZrCl_6_ (79% under 370 MPa)^38^, Li_6_PS_5_Cl (83% under 375 MPa)^39^, and Li_10_GeP_2_S_12_ (84.3% under 350 MPa)^40^. Therefore, although the LTC material was designed to be a positive electrode in the present study, its compressibility is rather competitive even in comparison with state-of-the-art inorganic solid-state electrolytes.

Now that LTC has been demonstrated to exhibit both of the two desired characteristics raised in the Introduction, i.e., high ionic conductivity and easy compressibility, whether this material can be reversibly cycled was examined via electrochemical measurements and analyses. As a chloride, LTC was found to be soluble in dimethyl carbonate (DMC), which is a common solvent of conventional non-aqueous liquid electrolyte solutions (Supplementary Fig. 2); this could be the reason why chlorides have rarely been investigated as electrode materials before^13,24^. However, with the liquid electrolyte replaced by the solid ones, this issue is circumvented. The investigation begins with cyclic voltammetry (CV), which was performed using an all-solid-state Li_13_Si_4_ | Li_6_PS_5_Cl (LPSCl) | Li_2_ZrCl_6_ (LZC) | LTC + C (weight ratio: LTC/C = 70/30) cell in the voltage range of 1.4–3.5 V *vs.* Li_13_Si_4_/Li^+^ at 0.5 mV s^–1^. According to the CV curve (Supplementary Fig. 3), the oxidation of Ti^3+^ to Ti^4+^ initiates at approximately 2.98 V *vs*. Li_13_Si_4_/Li^+^, while the subsequent reduction of Ti^4+^ to Ti^3+^ begins at 3.01 V *vs*. Li_13_Si_4_/Li^+^. As the scan continued, further reduction of Ti^3+^ to Ti^2+^ and oxidation of Ti^2+^ back to Ti^3+^ were also observed. These redox peaks are also present in the following cycles, and the peak positions remain unchanged. Therefore, the corresponding reactions seem reversible.

**Fig. 3:**
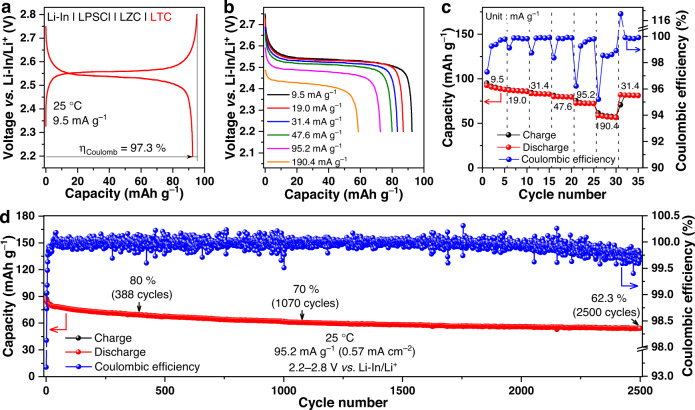
Electrochemical performance of the Li-In | LPSCl | LZC | LTC cell based on the Ti^3+^/Ti^4+^ redox couple. **a** The initial charge/discharge curves under 9.5 mA g^−1^ at 25 °C. **b**, **c** Rate capability at 9.5, 19.0, 31.4, 47.6, 95.2, and 190.4 mA g^−1^ at 25 °C. **d** Long-term cycling performance under 95.2 mA g^−1^ at 25 °C. All the cells were operated at 2.2−2.8 V *vs*. Li-In/Li^+^.

In light of this fact, we tested the charge and discharge behavior of the 300 °C-annealed LTC as the positive electrode in all-solid-state cells; the solid electrolyte is LZC (280 μm thick), and the negative electrode is Li-In alloy, which was separated from LZC by a layer of LPSCl (250 μm thick). It should be emphasized that the coexistence of two solid-electrolyte layers in the cell would inevitably compromise the energy density. Therefore, the cell configuration described above only serves the purpose of demonstrating the performance of the LTC positive electrode, but should not be adopted for practical high-energy batteries. In order to address this issue, appropriate surface coating needs to be developed for the chloride positive electrodes to eliminate the need for two thick, individual solid-electrolyte layers. Because the positive electrode active material here exhibits a rather high ionic conductivity beyond 1 mS cm^−1^ at 25 °C, no solid electrolyte was introduced into the positive electrode layer. Instead, only 5 wt% carbon black was added as the electronic conductive agents. As a result, 95 wt% of the composite positive electrode was occupied by the 300 °C-annealed LTC; such a mass fraction of positive electrode active materials surpasses that in all-solid-state cells based on oxide positive electrodes, which is usually below 80 wt% due to the need of over 20 wt% solid electrolyte to assist ion transport^27–32^. The Li-In | LPSCl | LZC | LTC cell with the high active-material fraction mentioned above achieved good battery performances at 25 °C under 1.5 tons. When cycled at 9.5 mA g^–1^ (95.2 mA g^–1^ = 1 C, corresponding to 1 Li per formula unit of LTC) between 2.2 and 2.8 V *vs*. Li-In/Li^+^, the cell shows an initial Coulombic efficiency of 97.3% and a discharge capacity of 92.5 mAh g^–1^ (Fig. 3a), which is 97.2% of the theoretical value for 1 Li per formula unit participating in the cycling (95.2 mAh g^−1^). Besides, the voltage for a given redox couple in this chloride positive electrode is higher than that in oxides. Specifically, the Ti^3+^/Ti^4+^ redox couple would usually give rise to a discharge voltage of around 1.5 V *vs*. Li/Li^+^ in oxides, as exemplified by Li_4_Ti_5_O_12_ and Li_0.33_La_0.56_TiO_3_^41,42^, but the same redox couple in LTC results in a higher discharge voltage of approximately 2.53 V *vs*. Li-In/Li^+^ (Fig. 3a and Supplementary Fig. 4). This fact entails that the crystal structure of LTC might favor high voltages^43^. If the Ti^3+^ in LTC is replaced by Ni^3+^, Mn^3+^, or Co^3+^, the discharge voltages higher than those in the present 4 V-class oxide positive electrodes might possibly be achieved. In addition to the initial capacity and voltage, the Li-In | LPSCl | LZC | LTC cell also delivers satisfactory rate capability. As shown in Fig. 3b, c, the average specific discharge capacities at 9.5, 19.0, 31.4, 47.6, 95.2, and 190.4 mA g^–1^ are 90.0, 86.4, 83.0, 79.7, 72.3, and 57.4 mAh g^–1^, respectively (in the present study, the specific currents and specific capacities are all calculated based on the mass of the active material in the positive electrode). Most importantly, excellent long-term cycling stability was achieved. As shown in Fig. 3d, when the LTC positive electrode was charged and discharged under 95.2 mA g^–1^ at 25 °C, it sustains over 80% capacity retention for as many as 388 cycles. Afterwards, the cycling behavior became even more stable. The capacity retention remained above 70% until the 1070th cycle. After 2500 cycles, a capacity retention of 62.3% with a final Coulombic efficiency of 99.7% was still achieved. Besides the cell based on the Li-In alloy negative electrode, the performance of the LTC positive electrode in the cell with the Li metal negative electrode was also examined. In order to alleviate the interfacial issues between Li metal and the sulphide solid electrolyte, a strategy reported recently by Li et al. was adopted: an Ag foil with a thickness of 1.3 μm was placed between LPSCl and Li metal to form the Li-Ag alloy and Ag-P-S-Cl protection layers^44^. The Li@Li-Ag | LPSCl | LZC | LTC cell assembled this way delivered an initial discharge capacity of 81.6 mAh g^–1^ with a Coulombic efficiency of 85.9% under 9.5 mA g^−1^ at 25 °C (Supplementary Fig. 5a), while relatively good rate capability (Supplementary Fig. 5b, c) and cycling stability (Supplementary Fig. 5d) were achieved as well. Considering that the electronic conductivity of LTC is orders of magnitude lower than its ionic conductivity, further improving the former could lead to even better cycling performance. Regardless, even with the present electronic conductivity, a rather low carbon content of 5 wt% in the composite positive electrode can already enable the decent cycling stability demonstrated above. The corresponding mass loading of active materials, i.e., 95 wt%, exceeded those typically needed for the oxide positive electrodes in all-solid-state cells (70−80 wt%^27–32^).

**Fig. 4:**
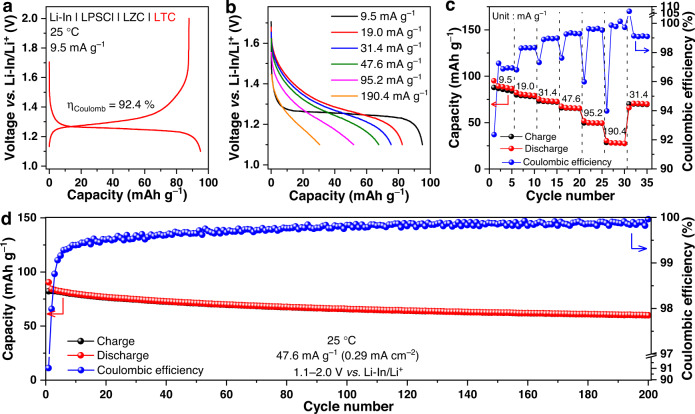
Electrochemical performance of the Li-In | LPSCl | LZC | LTC cell based on the Ti^2+^/Ti^3+^ redox couple. **a** The voltage profiles of the initial cycle under 9.5 mA g^−1^ at 25 °C. Since the discharge here takes place prior to the charge, which is opposite to other cells in the present study, the Coulombic efficiency *η*_Coulomb_ in this particular cell is defined as the ratio of charge capacity to discharge capacity. In this way, *η*_Coulomb_ would still be smaller than one, and thus can reflect the efficiency of cycling. **b**, **c** Rate capability at 9.5, 19.0, 31.4, 47.6, 95.2, and 190.4 mA g^−1^ at 25 °C. **d** Long-term cycling performance under 47.6 mA g^−1^ at 25 °C. All the cells were operated at 1.1−2.0 V *vs.* Li-In/Li^+^.

In addition to the Ti^3+^/Ti^4+^ redox couple, the Ti^2+^/Ti^3+^ one may also be utilized for reversible energy storage. When the cell above operated between 1.1 and 2.0 V *vs*. Li-In/Li^+^ at 25 °C with the cycling starting from the lithiation of LTC, an initial discharge capacity (95.1 mAh g^−1^) that is nearly identical with the theoretical value (95.2 mAh g^−1^) can be delivered at a specific current of 9.5 mA g^−1^ (Fig. 4a), with the corresponding voltage plateau located at 1.24 V *vs*. Li-In/Li^+^. The rate capability of the Ti^2+^/Ti^3+^ redox reaction in LTC appears lower to that of the Ti^3+^/Ti^4+^ reaction, but remains acceptable; as shown in Fig. 4b, c, the average specific discharge capacities at 9.5, 19.0, 31.4, 47.6, 95.2 and 190.4 mA g^–1^ are 89.5, 80.4, 73.7, 66.2, 50.1, and 28.4 mAh g^–1^, respectively. When being cycled at an intermediate specific current of 47.6 mA g^–1^, a decent cycling stability can be achieved too, as shown in Fig. 4d.

**Fig. 5:**
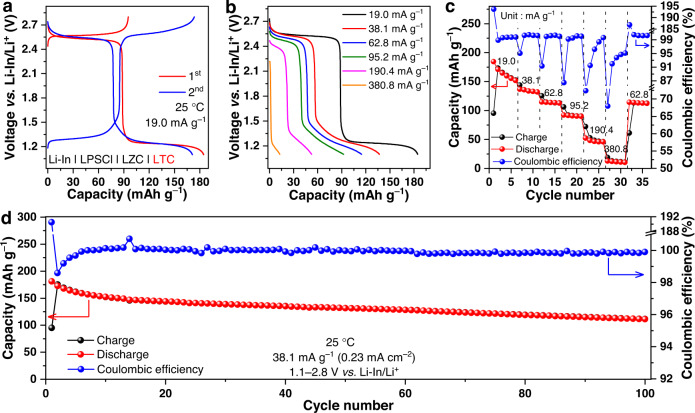
Electrochemical performance of the Li-In | LPSCl | LZC | LTC cell based on both the Ti^2+^/Ti^3+^ and Ti^3+^/Ti^4+^ redox couples. **a** The charge/discharge curves of the first two cycles under 19.0 mA g^−1^ at 25 °C. **b**, **c** Rate capability at 19.0, 38.1, 62.8, 95.2, 190.4 and 380.8 mA g^−1^ at 25 °C. **d** Long-term cycling performance under 38.1 mA g^−1^ at 25 °C. All the cells were operated at 1.1−2.8 V *vs*. Li-In/Li^+^.

Since the Ti^2+^/Ti^3+^ and Ti^3+^/Ti^4+^ redox reactions are both reversible, involving them altogether during cycling may enable the utilization of two Li per formula unit of LTC, and thus make the capacity doubled with respect to the values presented above. To explore this possibility, the Li-In | LPSCl | LZC | LTC cell was cycled in a broader voltage range of 1.1−2.8 V *vs*. Li-In/Li^+^. Figure 5a shows the charge and discharge curves of the first two cycles at 25 °C under 19.0 mA g^−1^. Consistent with the data in Figs. 3 and 4, the discharge voltage plateaux corresponding to the Ti^3+^/Ti^4+^ and Ti^2+^/Ti^3+^ redox reactions are clearly visible at 2.53 and 1.24 V *vs*. Li-In/Li^+^, respectively, while the occurrence of these reactions are corroborated by the differential capacity curves as well (Supplementary Fig. 6). With each voltage plateau contributing approximately one Li per formula unit of LTC, the initial discharge capacity under 19.0 mA g^−1^ at 25 °C reaches 184.5 mA g^−1^, which is 96.9% of the theoretical capacity for 2 Li per formula unit (190.4 mAh g^−1^). In the second cycle, both plateaux are still present, confirming that the nearly doubled capacity is reversible. Since the kinetics of Li-ion diffusion is relatively slow at the deeply lithiated state, cycling 2 Li per formula unit altogether cannot possibly exhibit the kind of high rate capability achieved by cycling only one of them through the Ti^3+^/Ti^4+^ redox couple (Fig. 3b, c). Regardless, a decent rate performance was still achieved; as shown in Fig. 5b, c, the average specific discharge capacities at 19.0, 38.1, 62.8, 95.2, 190.4, and 380.8 mA g^–1^ are 164.6, 134.3, 113.8, 91.0, 47.9, and 11.5 mAh g^–1^, respectively. More importantly, the large capacity arising from the two coexisting voltage plateaux also shows satisfactory stability during long-term cycling. After 100 cycles under 38.1 mA g^–1^ at 25 °C, a discharge capacity of 111.2 mAh g^–1^ with a Coulombic efficiency of 99.9% can still be delivered (Fig. 5d).

**Fig. 6:**
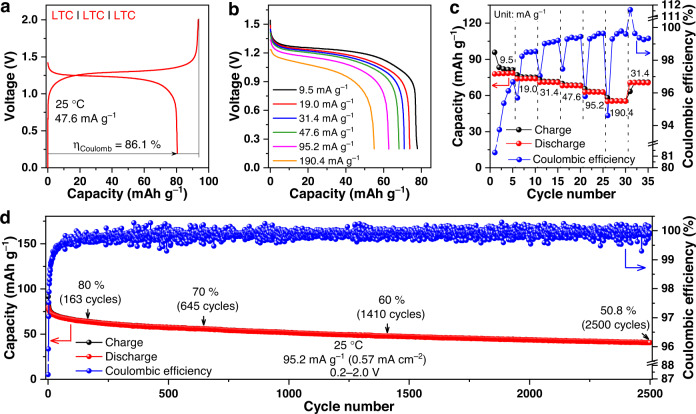
Electrochemical performance of the single-LTC cells. **a** The initial charge/discharge curves under 47.6 mA g^−1^ at 25 °C. **b**, **c** Rate capability at 9.5, 19.0, 31.4, 47.6, 95.2 and 190.4 mA g^−1^ at 25 °C. **d** Long-term cycling performance under 95.2 mA g^−1^ at 25 °C. All the cells were operated at 0.2−2.0 V.

While the results above clearly demonstrate that LTC is a valuable positive electrode active material, the electrochemical reversibility and relatively low voltage of its Ti^2+^/Ti^3+^ redox reaction (Fig. 4) suggest that this material may also acts as negative electrode active material. Additionally, because LTC shows high ionic conductivity and negligible electronic conductivity, it may serve as the solid electrolyte as well. Taking these characteristics into account, it seems that constructing a “single-LTC cell” with LTC being the positive electrode, negative electrode, and solid electrolyte simultaneously is feasible. An attempt was thus made to verify this possibility. Since the rate capability of the Ti^2+^/Ti^3+^ redox reaction is inferior to that of the Ti^3+^/Ti^4+^ one (Fig. 3c and Fig. 4c), the single-LTC cell in the present study was assembled with a relatively high negative/positive capacity ratio of 3. Figure 6a displays the initial charge and discharge curves of this cell at 25 °C under 47.6 mA g^−1^. With the discharge voltage plateau located at approximately 1.24 V (Fig. 6a and Supplementary Fig. 7), the cell delivers an initial discharge capacity of 80.5 mAh g^–1^ (the mass refers to that of the positive electrode) and a Coulombic efficiency of 86.1%. The rate capability is also satisfactory; as shown in Fig. 6b and c, the average specific discharge capacities at 9.5, 19.0, 31.4, 47.6, 95.2, and 190.4 mA g^–1^ are 78.2, 73.9, 70.9, 68.1, 62.8 and 55.2 mAh g^−1^ of the positive electrode, respectively. Last but not least, decent long-term cycling stability was achieved as well. The capacity retention was above 80% for 163 cycles, and maintained at 50.8% after 2500 cycles (Fig. 6d). During such long-term cycling, the Coulombic efficiency was also rather stable, and a high value of 99.9% was sustained at the 2500th cycle. The cycling performance presented above extends the number of cycles limit compared with the state-of-the-art single-material cells, e.g., the ones based on Li_10_GeP_2_S_12_^26^ and Li_1.5_Cr_0.5_Ti_1.5_(PO_4_)_3_^45^, whose maximum reported cycling number never exceeds 10. It should be noted that, although the particle size and morphology are known to significantly influence the performance of the positive electrode, the cell performance presented above was achieved without any optimization in this regard. Instead, the LTC particles used as the active materials here were synthesized using the mechanochemical approach, which may only result in non-uniform particle size and morphologies (Supplementary Fig. 8). If these characteristics are tailored using the synthesis methods suitable for such a purpose, e.g., the ammonium-assisted wet chemistry^31^ and solid-state reaction^30^, the performance even better than that reported here may be expected.

**Fig. 7:**
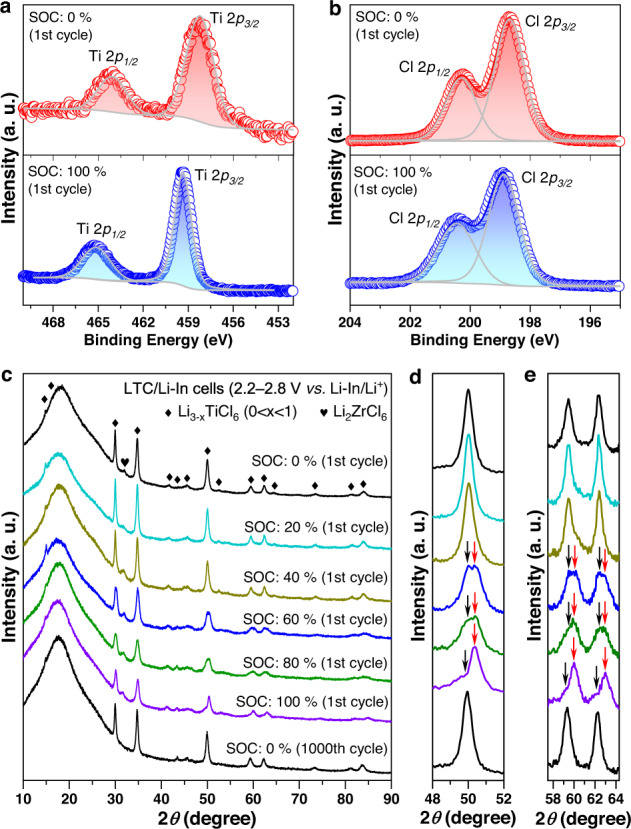
Li-ion storage mechanism of the LTC positive electrode based on the Ti^3+^/Ti^4+^ redox couple. **a**, **b** Ex situ Ti-2*p* (**a**) and Cl-2*p* (**b**) X-ray photoelectron spectra of the LTC positive electrode in the Li-In | LPSCl | LZC | LTC cell at different SOCs of its initial charge (the corresponding voltage profile with SOCs indicated is displayed in Supplementary Fig. 9). **c** Ex situ XRD patterns of the LTC positive electrode from the Li-In | LPSCl | LZC | LTC cells at different SOCs. **d**, **e** Close-up views of the XRD patterns in **c**. The data for each SOC is plotted in the same color as that in **c**. In the XRD patterns for 60−100% SOCs of the 1st cycle, the peaks of the original phase and those from the new phase are arrowed in black and red, respectively.

**Fig. 8:**
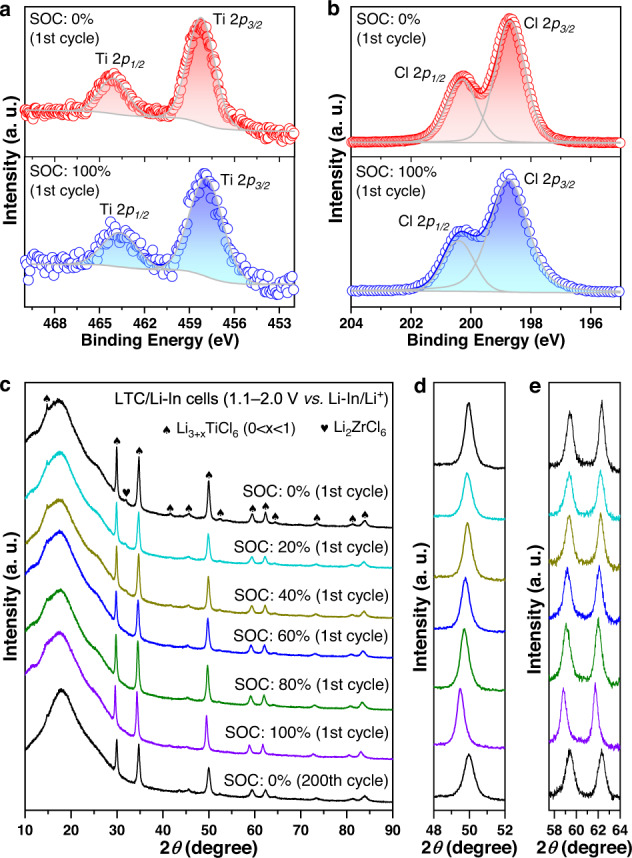
Li-ion storage mechanism of the LTC positive electrode based on the Ti^2+^/Ti^3+^ redox couple. **a**, **b** Ex situ Ti-2*p* (**a**) and Cl-2*p* (**b**) X-ray photoelectron spectra of the LTC positive electrode in the Li-In | LPSCl | LZC | LTC cell at different SOCs of its initial discharge (the corresponding voltage profile with SOCs indicated is displayed in Supplementary Fig. 11). **c** Ex situ XRD patterns of the LTC positive electrode from the Li-In | LPSCl | LZC | LTC cells at different SOCs. **d**, **e** Close-up views of the XRD patterns in **c**. The data for each SOC is plotted in the same color as that in **c**.

With the appealing characteristics of the LTC positive electrode demonstrated above, its Li-ion storage mechanism was explored. The study began with the investigation on the delithiation of LTC, i.e., the procedure happening through the Ti^3+^/Ti^4+^ redox couple. To this end, ex-situ X-ray photoelectron spectroscopy (XPS) and XRD measurements were performed at different stages of the initial charging cycle for the Li-In | LPSCl | LZC | LTC cell under 9.5 mA g^−1^ between 2.2−2.8 V *vs*. Li-In/Li^+^ at 25 °C; the corresponding voltage profile with the states of charge (SOCs) indicated is displayed in Supplementary Fig. 9. The XPS data of LTC at 0% and 100% SOCs of the aforementioned cell are compared in Fig. 7a, b. After delithiation, the Ti-2*p* spectrum shifts to higher energies, while Cl-2*p* undergoes no noticeable variation. Therefore, the charge balance after the removal of Li^+^ should be maintained by the oxidation of Ti^3+^, consistent with the CV results in Supplementary Fig. 3. With the charge compensation mechanism confirmed by XPS, the structural evolution was explored. Figure 7c shows the XRD patterns of LTC when the cell in Supplementary Fig. 9 was charged to different stages. With the ex situ XRD measurement performed directly on the surface of the all-solid-state cell, the Li_2_ZrCl_6_ solid electrolyte lying below the positive electrode layer would inevitably contribute minor signals, but the evolution of LTC remains clearly visible. At SOCs below 60%, the delithiation of LTC does not result in any phase transition; only the diffraction peaks gradually shift toward high angles (Fig. 7c), indicating that the unit cell is shrinking. Therefore, the delithiation at this stage should occur through a deintercalation mechanism without fundamentally altering the crystal structure. With further delithiation to SOCs beyond 60%, this deintercalation mechanism is replaced by the phase-transition mechanism: the continuous removal of Li induces the emergence of another set of diffraction peaks (examples arrowed in red in Fig. 7d, e) at angles slightly above the original ones (arrowed in black in Fig. 7d, e). As delithiation proceeds, the original set of diffraction peaks, i.e., the ones located at relatively low angles, no longer undergoes any position shifting. Instead, their intensities keep decreasing with respect to the new set of diffraction peaks. At 100% SOC, the latter almost dominates the entire XRD pattern. According to this observation, the delithiation at SOCs above 60% must occur through a mechanism similar to that between Li_4_Ti_5_O_12_ and Li_7_Ti_5_O_12_^46,47^. That is, instead of gradually removing Li from a largely unchanged atomic framework, delithiation occurs through the emergence and growth of a relatively Li-deficient phase with a crystal structure similar to that of the original one. The precise structural determination of this Li-deficient phase is challenging, as it requires a sample that is free from the original LTC phase. Regardless, the emergence of the phase-transition mechanism following deintercalation does not compromise the reversibility of this material. Even after undergoing 1000 cycles of delithiation and lithiation, the LTC at 0% SOC remains in a single phase with the original *C2/m* structure (Fig. 7c−e). Besides, such prolonged cycling did not result in significant decrease of the crystallite size either, as reflected by the nearly unchanged width of diffraction peaks (Supplementary Fig. 10). Therefore, the crystallinity should be largely preserved, without any considerable degradation after repeated cycling. Beyond the reaction associated with the Ti^3+^/Ti^4+^ redox couple, a similar study was conducted to that based on the Ti^2+^/Ti^3+^ redox couple as well. Specifically, ex-situ XPS and XRD measurements were performed at different stages during the lithiation of LTC, which was realized by discharging a Li-In | LPSCl | LZC | LTC cell under 9.5 mA g^−1^ between 1.1 and 2.0 V *vs*. Li-In/Li^+^ at 25 °C (Supplementary Fig. 11). While the XPS data confirm that the lithiation took place through the redox reaction of Ti, instead of Cl (Fig. 8a and b), the ex-situ XRD patterns do not indicate the emergence of any additional phase during the entire procedure. Instead, only a shifting of the diffraction peaks toward low angles were observed, and such a change is highly reversible after 200 cycles of lithiation and delithiation (Fig. 8c−e). Therefore, the procedure associated with the Ti^2+^/Ti^3+^ redox couple in LTC should be based upon an intercalation mechanism.

Although the voltage and capacity of LTC have not yet been able to rival the state-of-the-art layered oxide positive electrode active materials, its discovery points out that the transition-metal chlorides are very promising candidates for the positive electrodes in all-solid-state batteries. The reason is at least three-fold. First of all, in terms of the specific energy with respect to the mass of active materials (equal to the product between the average cell discharge voltage and the specific capacity of the active material^3,48^), the transition-metal chloride positive electrode reported here has in fact outperformed the oxides based on the same transition-metal element. In LTC, the potential for the Ti^3+^/Ti^4+^ redox couple (2.53 V *vs*. Li-In/Li^+^) is more than two times that in most Ti-based oxides (around 1.5 V *vs*. Li/Li^+ ^^42,49^). Consequently, the calculated specific energy associated with this redox couple reaches 299.9 Wh kg^−1^ (95.2 mAh g^−1^ × 3.15 V *vs*. Li/Li^+^; since the Li-In electrode shows a voltage plateau at 0.62 V *vs*. Li/Li^+ ^^50^, converting the aforementioned 2.53 V *vs*. Li-In/Li^+^ potential into the value *vs*. Li/Li^+^ would yield 2.53 V + 0.62 V = 3.15 V), while that for Li_4_Ti_5_O_12_, as a representative Ti-based oxide electrode, is only 262.5 Wh kg^−1^ (175.0 mAh g^−1^  × 1.5 V *vs*. Li/Li^+ ^^42,49^). Besides, the Ti^2+^/Ti^3+^ redox reaction in LTC also exhibits a relatively high potential of 1.24 V *vs*. Li-In/Li^+^, along with a decent reversibility, while in Ti-based oxide electrode active materials such a low-valence redox couple is barely utilized due to the overly low potential and/or poor reversibility. The Ti^2+^/Ti^3+^ redox couple in LTC contributes an additional calculated specific energy of 177.1 Wh kg^−1^ (95.2 mAh g^−1^ × 1.86 V *vs*. Li/Li^+^; since the Li-In electrode shows a voltage plateau at 0.62 V *vs*. Li/Li^+ ^^50^, converting the aforementioned 1.24 V *vs*. Li-In/Li^+^ potential into the value *vs*. Li/Li^+^ would yield 1.24 V + 0.62 V = 1.86 V), so the total calculated specific energy associated with the reversible redox couples is 477.0 Wh kg^−1^ for LTC, higher than that of Li_4_Ti_5_O_12_ (262.5 Wh kg^−1^). If such advantages over the mentioned state-of-the-art Ti-based oxide electrode active materials are inherited by the transition-metal chlorides based on Ni, Mn, and/or Co, they could maybe surpass the present 4 V-class oxide positive electrodes such as LiCoO_2_ and LiNi_0.8_Mn_0.1_Co_0.1_O_2_ on energy content. Secondly, the high ionic conductivity of the transition-metal chlorides eliminates the need of solid electrolytes in the composite positive electrode, and consequently allows for a rather high mass loading of the active materials such as 95 wt%. In contrast, the state-of-the-art layered oxide positive electrodes need to be mixed with a considerable amount of solid electrolytes, leading to an active material weight content of only 70−80 wt%^27–32^. This difference would make the high energy density more easily achievable for the cells based on transition-metal chloride-positive electrodes. Last but not least, as mentioned in Introduction, the compressibility of the chlorides may ensure an intimate solid-solid contact under external pressure even after the repeated cycling leads to cracks in the particles, while this is very challenging to achieve for the brittle oxides.

## Discussion

In summary, we report a highly Li-ion conductive and easily compressible positive electrode that is suitable for all-solid-state batteries. Described by the chemical formula Li_3_TiCl_6_, it exhibits a layered structure with the *C*2/*m* space group. Differently from standard layered oxide positive electrode active materials such as LiCoO_2_, the transition-metal sites of this layered chloride are not fully occupied and allow Li ions to migrate through. Therefore, its atomic framework is effective not only in Li-ion storage, but also in ion transport; the 300 °C-annealed LTC exhibits a rather high ionic conductivity of 1.04 mS cm^–1^ at 25 °C. Combined with the good compressibility, such a high ionic conductivity avoid the use of solid electrolytes in the composite positive electrode, and in turn enables a high active-material fraction of 95 wt%, which is higher than those for typical ASSLBs in literature (usually below 80 wt%^27–32^). More importantly, this composite positive electrode delivers excellent performance in all-solid-state cells. When the Li-In | LPSCl | LZC | LTC cell was cycled under 95.2 mA g^−1^ at 25 °C, the capacity retention was above 80% for 388 cycles, and maintained at 62.3% after 2500 cycles. Besides, the single-material cell with LTC simultaneously serving as the positive electrode, negative electrode, and solid electrolyte also exhibited a decent cycle life (capacity retention above 80% and 60% after 163 and 1410 cycles, respectively, under 95.2 mA g^−1^ at 25 °C) that exceeds those of other single-material cells in literature (no more than 10 cycles reported)^26,45^. These performances suggest that the transition-metal chlorides, albeit barely studied as positive electrodes before due to their high solubility in liquid electrolytes, are in fact highly promising candidates for the positive electrodes in ASSLBs.

## Methods

### Materials synthesis

Li_3_TiCl_6_ was synthesized from stoichiometric amount of LiCl (Alfa Aesar, 99.9%) and in-house developed TiCl_3_, which was obtained by purifying the commercial TiCl_3_–AlCl_3_ (Alfa Aesar, TiCl_3_ 76.0–78.5%). The purification of TiCl_3_–AlCl_3_ is realized by sealing the chemical in a vacuum quartz tube and then annealing it at 180 °C, 200 °C, and 300 °C, subsequently. The annealing at each temperature lasted for 24 h, and a fresh quartz tube was used at each annealing temperature. During the entire purification procedure, the powders had been exposed to Ar only, and air exposure was strictly avoided. The phase-purity of the TiCl_3_ acquired this way was confirmed by X-ray diffraction (Supplementary Fig. 12). The synthesis of Li_3_TiCl_6_ began with milling the stoichiometric amount of raw materials in the Ar atmosphere within a Si_3_N_4_ pot with ZrO_2_ balls (ball-to-powder mass ratio of 12:1) in a planetary mill (FRITSCH, Pulverisette 7 premium line) at 600 rpm for 24 hours. The obtained material was referred to as the “as-milled LTC”. After the “as-milled LTC” was sealed in a vacuum quartz tube and annealed at 300 °C for 5 hours, the “300 °C-annealed LTC” in the main text was acquired. The Li_2_ZrCl_6_ (LZC) material used for assembling ASSLBs was synthesized mechanochemically from LiCl (Alfa Aesar, 99.9%) and ZrCl_4_ (Acros Organics BVBA, 98%). The milling parameters for synthesizing LZC are largely the same as those for the “as-milled LTC”; the only difference is the milling time, which is 45 h for LZC.

### Structure and compressibility characterization

X-ray diffraction (XRD) was performed using a Rigaku Ultima IV diffractometer with Cu K*α*1 radiation, and the powders were sealed in Kapton film (conducted within an Ar-filled glovebox with both the H_2_0 and O_2_ contents no higher than 0.01 ppm) to avoid air exposure. Rietveld refinement was performed using GSAS II^33,34^. During refinement, the overall Li:Ti:Cl atom ratio was kept at 3:1:6. The BVSE analysis was conducted using the softBV program^35,36^; the energy profiles of the Li-ion transport pathways were calculated against a 3D grid of points with 0.1 Å resolution using the transferable Morse-type softBV force field. The X-ray photoelectron spectroscopy (XPS) data were collected on a Thermo Scientific K-Alpha spectrometer using monochromatic Al Kα radiation (1486.6 eV). The Li-In | LPSCl | LZC | LTC cells used for the ex-situ XRD and XPS measurements were assembled with the same approach and parameters (including the mass loading of the active material, amount of the solid electrolytes, pressure, etc.) as those used for the cycling tests. After the SOC for study was reached, the pelletized cell was removed from the PEEK mould as a whole, and then the XRD or XPS measurement was conducted on the LTC side of this pellet. For the ex-situ XRD measurement, the air exposure during specimen transfer was prevented by sealing the pelletized cell using Kapton film in an Ar-filled glove box with both the H_2_0 and O_2_ contents no higher than 0.01 ppm. For the ex-situ XPS measurement, this was realized by using a vacuum transfer holder associated with the instrument. The scanning electron microscopy (SEM) observation was conducted using a ZEISS GeminiSEM 500 microscope; the secondary electron imaging and the energy-dispersive X-ray spectroscopy (EDS) were performed under the acceleration voltages of 5 and 10 kV, respectively. In order to determine the relative density of the cold-pressed LTC pellet, 100 mg of 300 °C-annealed LTC powder was first pressed in a mould with 6 mm inner diameter under 350 MPa for 2 minutes. After removing the pellet from the mould, its thickness, diameter, and weight were measured to calculate the actual density. The relative density of this cold-pressed pellet was determined by comparing its actual density with the theoretical density of LTC (2.266 g cm^−3^, as determined by Rietveld refinement).

### Conductivity measurements

The electrochemical impedance spectroscopy (EIS) measurements were performed on the cold-pressed powder under an external pressure of 380 MPa using a MTZ-35 impedance analyzer (Bio-Logic). The frequency range was between 1 Hz and 35 MHz. The potential driving amplitude was 50 mV. The electronic conductivity was determined by direct current (DC) polarization measurement of the cold-pressed powder under 380 MPa using an electrochemical workstation (CHI630E, CH Instruments, Inc.) with an applied voltage of 1 V.

### Electrochemical characterizations

The all-solid-state cells were assembled in an Ar-filled glovebox (water and oxygen contents both below 0.01 ppm) using the mould shown in Supplementary Fig. 13. The cyclic voltammetry (CV) measurement was performed on Li_13_Si_4_ | LPSCl | LZC | LTC + C (weight ratio: LTC/C = 70/30) cells between 1.4 and 3.5 V at 0.5 mV s^–1^ (Li_13_Si_4_ was purchased from WOWMATERIS, Changzhou). For the Li-In | LPSCl | LZC | LTC, Li@Li-Ag | LPSCl | LZC | LTC, and single-LTC cells, the LTC composite electrodes were prepared by mixing the 300 °C-annealed LTC powder with carbon black (Super P, Shenzhen Kejing Star Technology Company, China) in an Ar-filled glovebox with both the H_2_0 and O_2_ contents no higher than 0.01 ppm using a vortex mixer (Haimen Kylin-Bell Lab Instruments, QL-866) for 10 minutes in a weight ratio of 95:5. The assembly of the Li-In | LPSCl | LZC | LTC cell began with pressing 60 mg of the LZC powder in a PEEK mould with an inner diameter of 10 mm under 0.6 tons. Then, 40 mg LPSCl (99%, Shenzhen Kejing Star Technology Company) powder was spread to one side of the LZC layer and pressed at 1 ton, followed by sprinkling and pressing 5 mg of the LTC composite electrode powder on the other side of the LZC layer at 3 tons. Finally, a piece of In foil (100 μm thick, 10 mm diameter, 99.995%, Qinghe County Chengshuo Metal Materials Co., Ltd) and a piece of Li foil (50 μm thick, 10 mm diameter, 99.9%, China Energy Lithium Co., Ltd) were attached successively to the LPSCl layer under the pressure of 1.5 tons. According to the SEM observation, the thicknesses of the positive electrode layer, the LZC layer, and the LPSCl layer in the cell fabricated this way are 35, 280, and 250 μm, respectively (Supplementary Fig. 14). The Li@Li-Ag | LPSCl | LZC | LTC cell was assembled in the same manner, except that the In foil was replaced by a piece of Ag foil (1.3 μm thick, 10 mm diameter, 99.99%, Nanjing Yongbo Metal Materials Co., LTD) and the final pressure was 0.2 tons. As for the single-LTC cell, The solid-electrolyte layer was formed first by pressing 60 mg of the 300 °C-annealed LTC powder in the aforementioned PEEK mould at 0.6 tons. Then, 5 and 15 mg of the LTC composite electrode powder prepared above were sprinkled on both sides of the solid-state electrolyte pellet and pressed under 3 tons to serve as the positive and negative electrode, respectively. The thicknesses of the positive electrode layer, solid-electrolyte layer, and the negative electrode layer in such single-LTC cells are 35 μm, 250 μm, and 80 μm, respectively (Supplementary Fig. 15). The Li-In | LPSCl | LZC | LTC, Li@Li-Ag | LPSCl | LZC | LTC, and the single-LTC cells were cycled under an external pressure of 1.5, 0.2, and 1.5 tons, respectively, at 25 ± 1 °C using a battery testing system (CT2001A, Wuhan LAND Electronic Co. Ltd). All the electrochemical tests were conducted at a temperature of 25 ± 1 °C, which was ensured by placing the cells in an incubator (Tianjin Hongnuo Instrument, SPX-250B, temperature accuracy ±1 °C). For each electrochemical experiment, at least 10 cells were tested to ensure the reproducibility of the results. All the specific currents and specific capacities reported in the present study were calculated based on the mass of the active material in the positive electrode.

## Supplementary information

Supplementary Information

## Data Availability

The data that support the findings of this study are available within the article (and its Supplementary Information files) and from the corresponding authors upon reasonable request.
