# Supplementary material for: Li3TiCl6 as ionic conductive and compressible positive electrode active material for all-solid-state lithium-based batteries
Source: Nat Commun. 2023 Mar 13;14:1396. doi: 10.1038/s41467-023-37122-7 (PMC10011600; doi:10.1038/s41467-023-37122-7)
Supplement: Supplementary file 1 — Supplementary Information [file 41467_2023_37122_MOESM1_ESM.pdf]

Supplementary Information

Li_3TiCl_6 as ionic conductive and compressible positive electrode active material for all-solid-state lithium-based batteries

Kai Wang[1,2], Zhenqi Gu[1], Zhiwei Xi[1], Lv Hu[1] and Cheng Ma[1,3]*

[1]Hefei National Research Center for Physical Sciences at the Microscale, CAS Key Laboratory of Materials for Energy Conversion, Department of Materials Science and Engineering, University of Science and Technology of China, Hefei, Anhui 230026, China.

[2]School of Materials & Energy, Lanzhou University, Lanzhou, Gansu 730000, China.

[3]National Synchrotron Radiation Laboratory, Hefei, Anhui 230026, China.

*Corresponding author: mach16@ustc.edu.cn

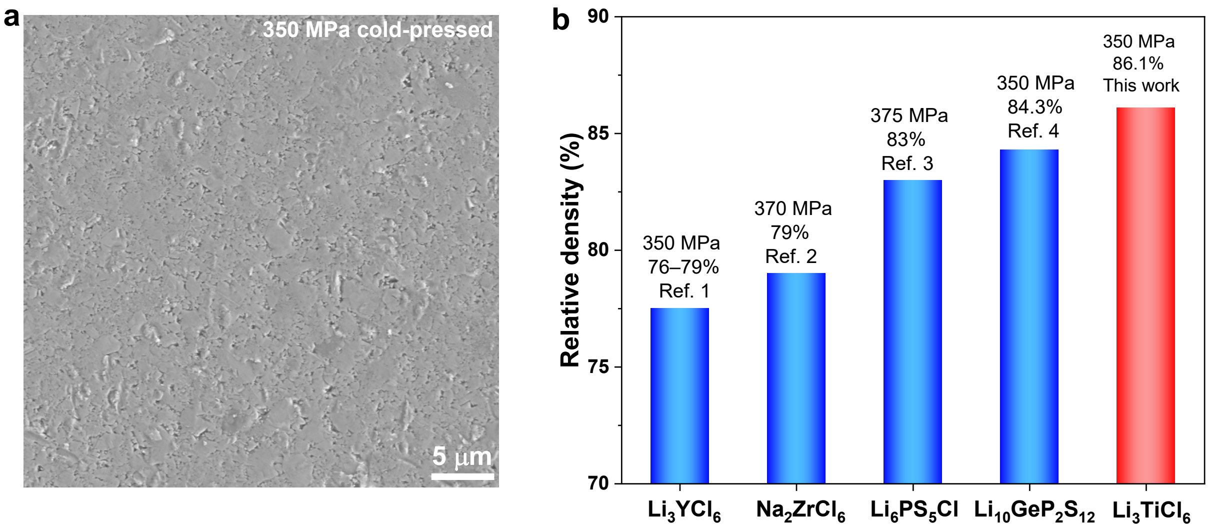

Supplementary Figure 1. **a** SEM image for the surface of the pellet fabricated by cold pressing the 300 °C-annealed LTC powder under 350 MPa. **b** The relative density of the cold-pressed pellet of 300 °C-annealed LTC and those of the cold-pressed pellets of several widely studied compressible solid electrolytes[1-4]. The pressures used for fabricating the pellets are all denoted.

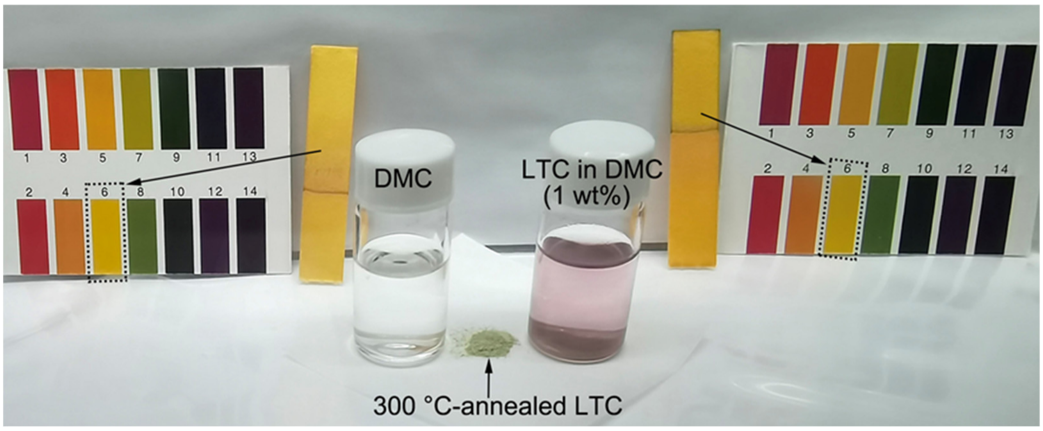

Supplementary Figure 2. Solubility test of 300 °C-annealed LTC. After 43mg 300 °C-annealed LTC was poured into 4 mL dimethyl carbonate (DMC), the former completely dissolved. During this procedure, the originally colorless solvent (left) became a pink solution (right). The solubility test was conducted in an Ar-filled glovebox (water and oxygen contents both below 0.01 ppm) at 25 °C.

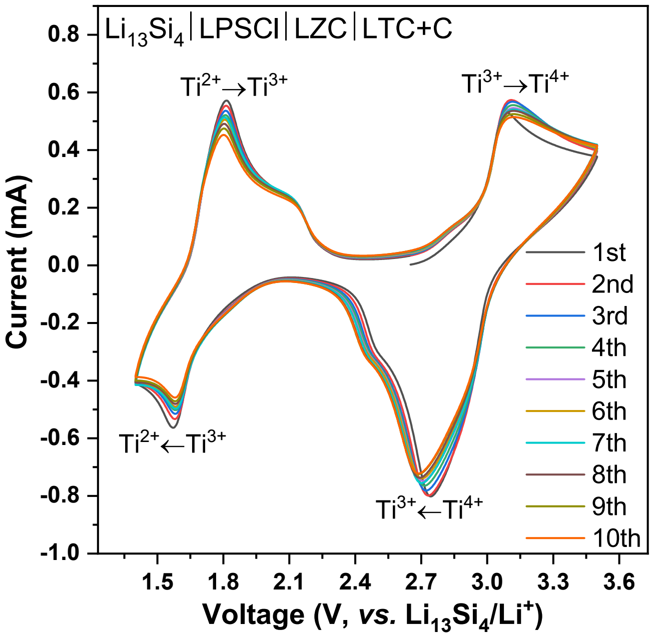

Supplementary Figure 3. CV curves of the $Li_{13}Si_4$ | LPSCl | LZC | LTC+C cell at 0.5 mV s^{-1} and 25 °C.

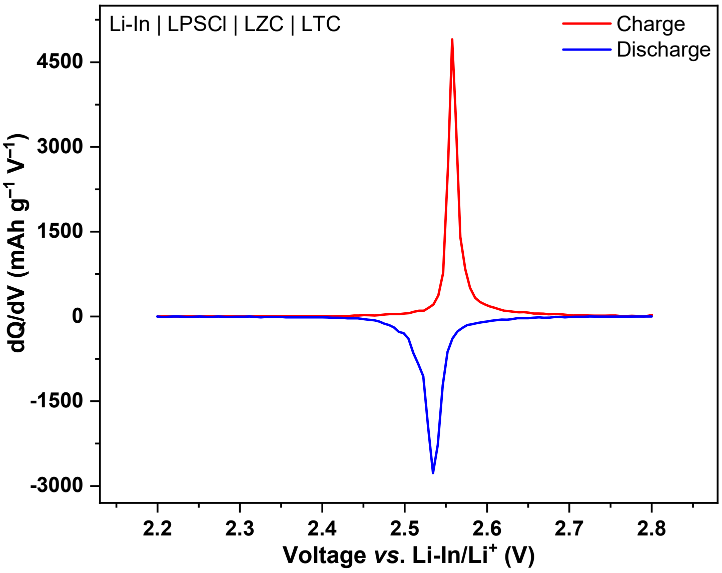

Supplementary Figure 4. Differential capacity against voltage (dQ/dV) curves corresponding to the initial charge/discharge curves for the Li-In | LPSCl | LZC | LTC cell shown in Figure 3a of the main text.

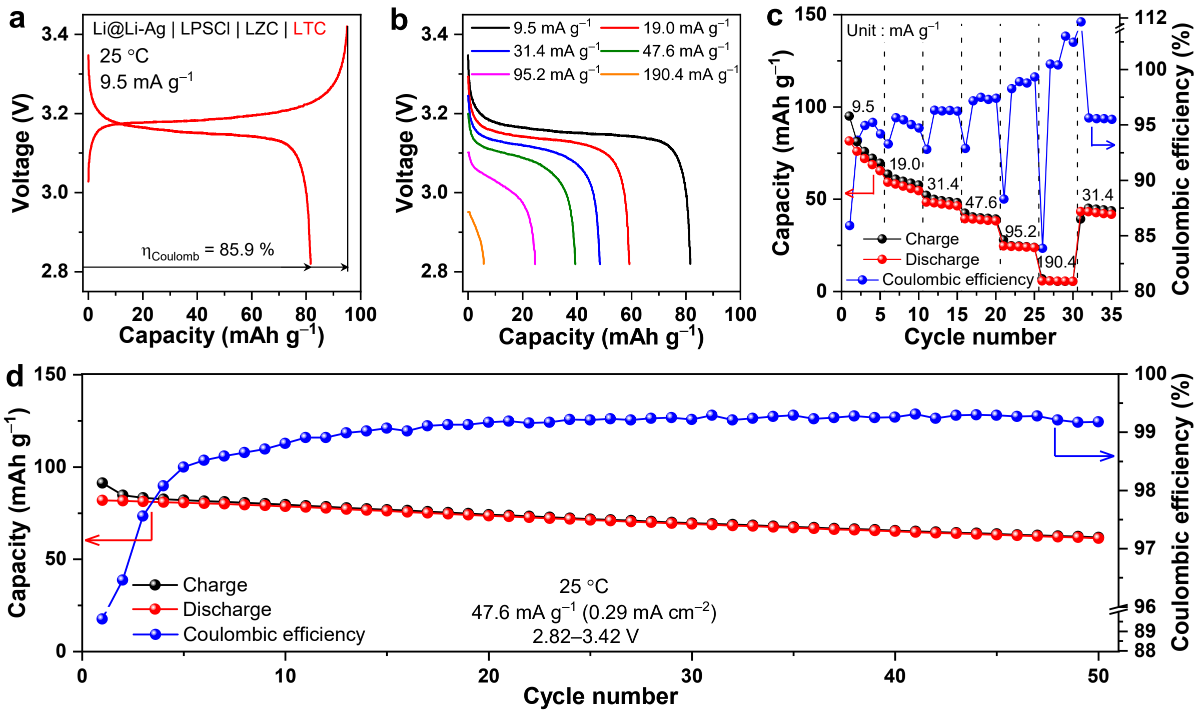

Supplementary Figure 5. Electrochemical performance of the Li@Li-Ag | LPSCl | LZC | LTC cell. **a** The initial charge/discharge curves under 9.5 mA g^{-1} at 25 °C. **b**, **c** Rate capability at 9.5, 19.0, 31.4, 47.6, 95.2 and 190.4 mA g^{-1} at 25 °C. **d** Long-term cycling performance under 47.6 mA g^{-1} at 25 °C. All the cells were operated at 2.82–3.42 V vs. Li/Li^+.

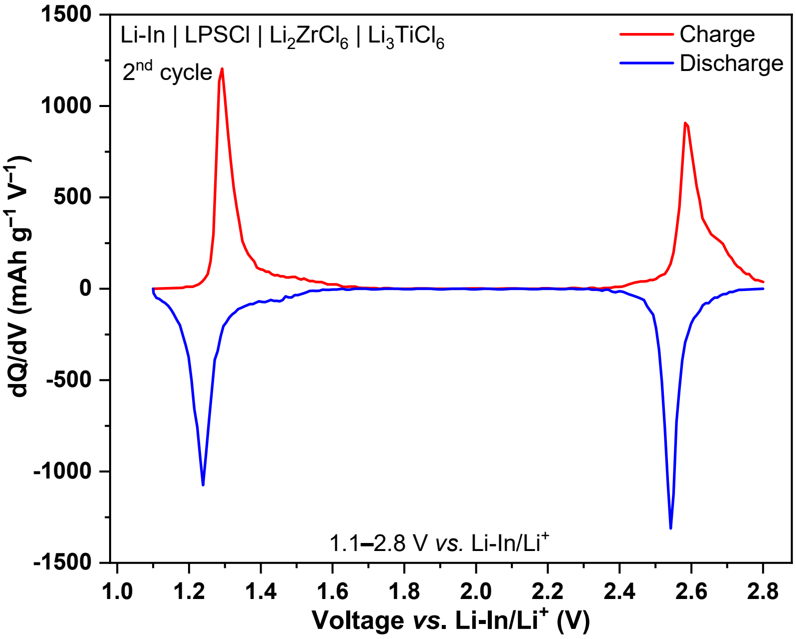

Supplementary Figure 6. Differential capacity against voltage (dQ/dV) curves corresponding to the charge/discharge curves of the 2nd cycle for the Li-In | LPSCl | LZC | LTC cell shown in Figure 5a of the main text.

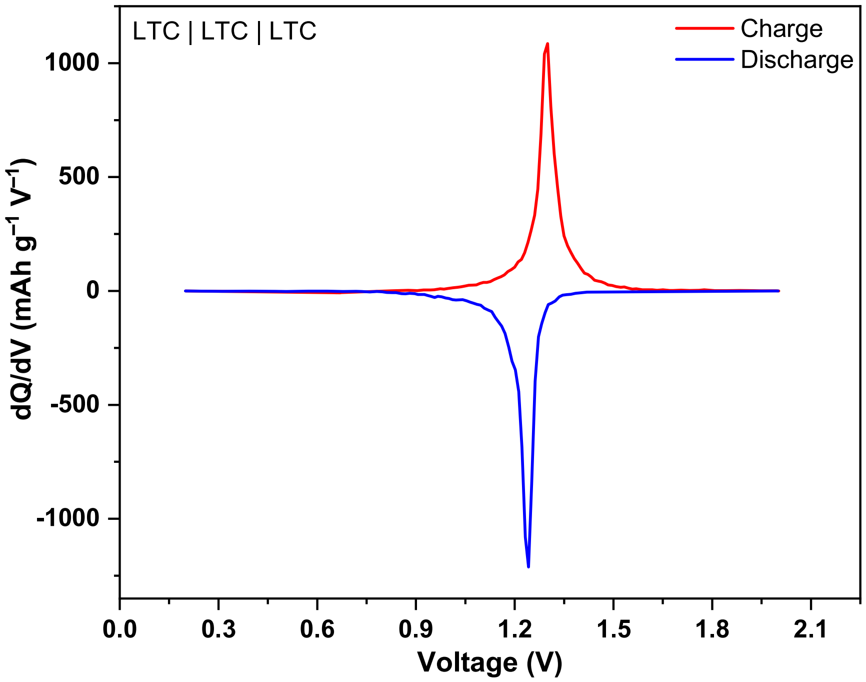

Supplementary Figure 7. Differential capacity against voltage (dQ/dV) curves corresponding to the initial charge/discharge curves for the single-LTC cell shown in Figure 6a of the main text.

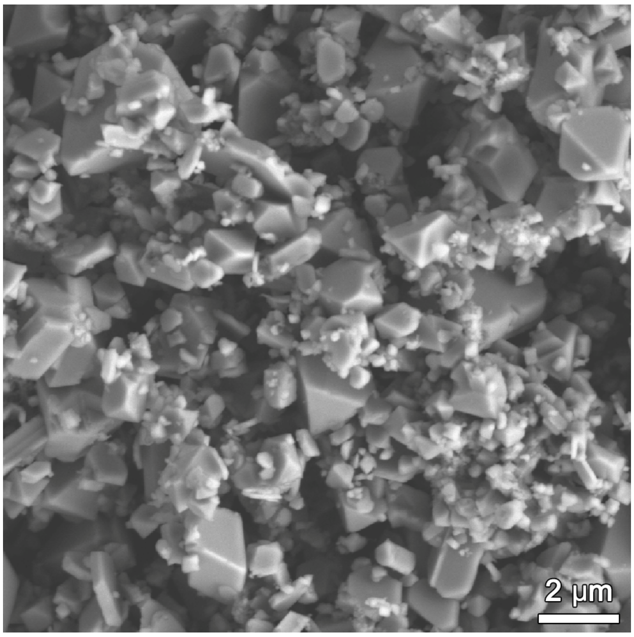

Supplementary Figure 8. SEM images of the 300 °C-annealed LTC powder.

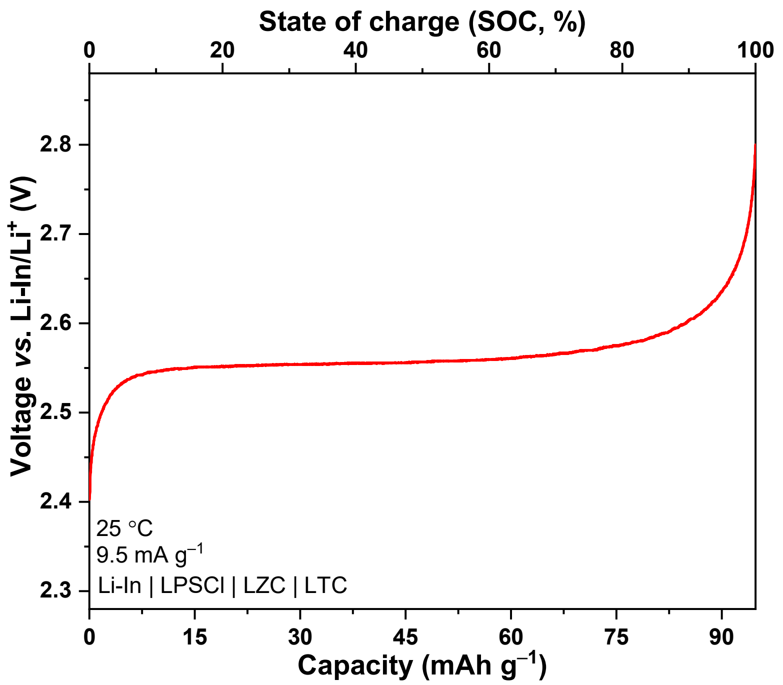

Supplementary Figure 9. The initial charge curve of the Li-In | LPSCl | LZC | LTC cell (2.2–2.8 V *vs.* Li-In/Li$^+$) under 9.5 mA g^{-1} at 25 °C, with the states of charge (SOCs) indicated.

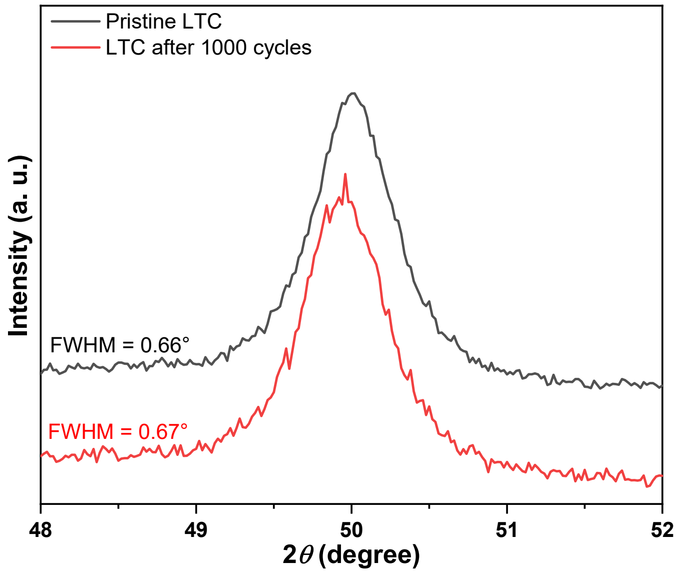

Supplementary Figure 10. The full widths at half maximum (FWHMs) of the $(33\bar{1})$ X-ray diffraction peak of the pristine Li_3TiCl_6 and that of Li_3TiCl_6 after 1000 cycles under 95.2 mA g^{-1} at 25 °C. Both Li_3TiCl_6 samples were analysed as electrodes, rather than powders.

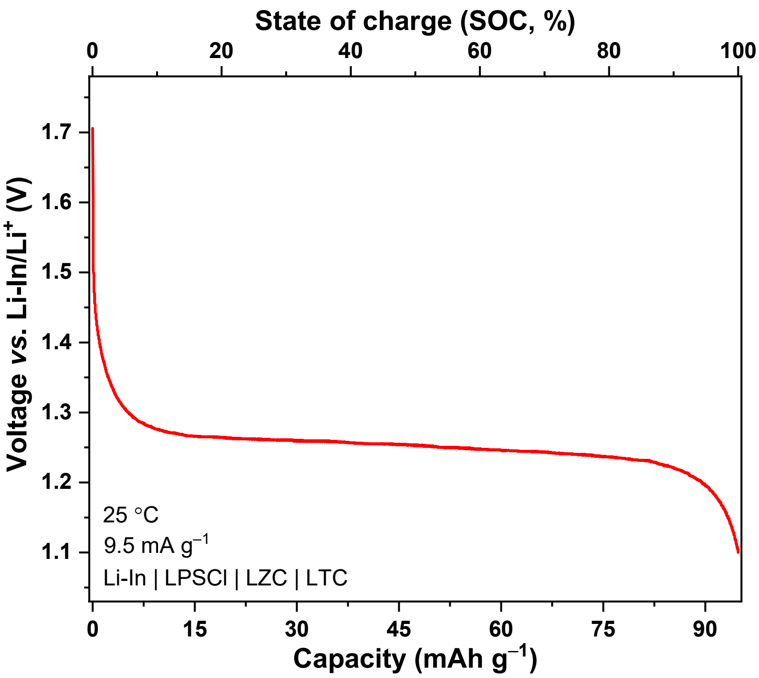

Supplementary Figure 11. The initial discharge curve of the Li-In | LPSCl | LZC | LTC cell (1.1–2.0 V *vs.* Li-In/Li^+) under 9.5 mA g^{-1} at 25 °C, with the states of charge (SOCs) indicated.

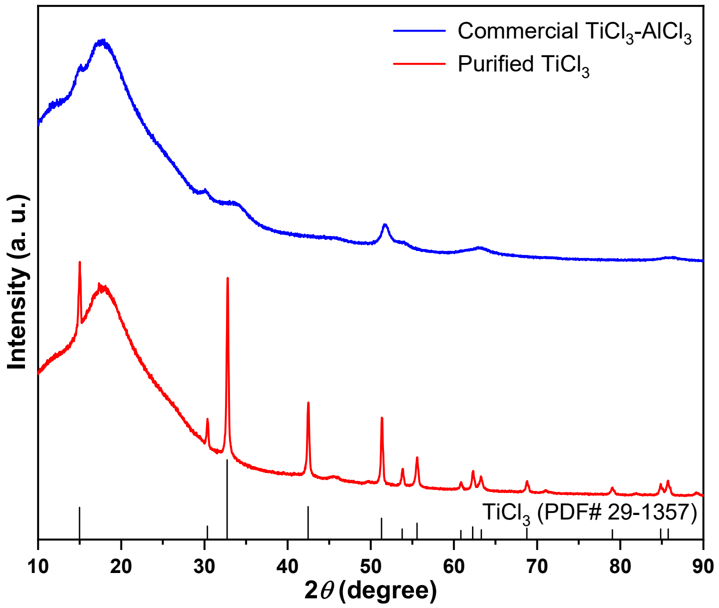

Supplementary Figure 12. XRD patterns of the commercial $TiCl_3$-$AlCl_3$ (Alfa Aesar, $TiCl_3$ 76.0–78.5%) and the $TiCl_3$ acquired by purifying the former. Detailed purification procedures were described in Methods.

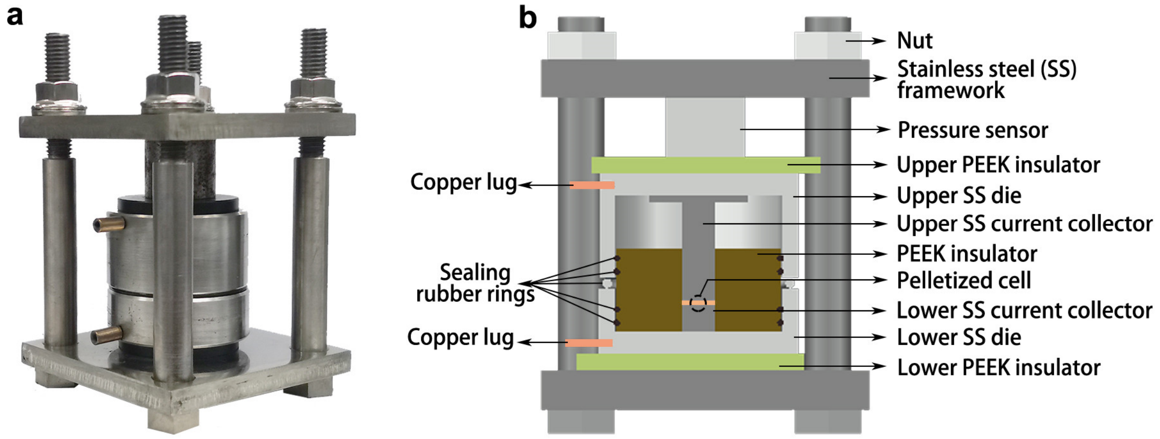

Supplementary Figure 13. The photograph (**a**) and schematic illustration (**b**) of the mould used for assembling the all-solid-state cells in the present study.

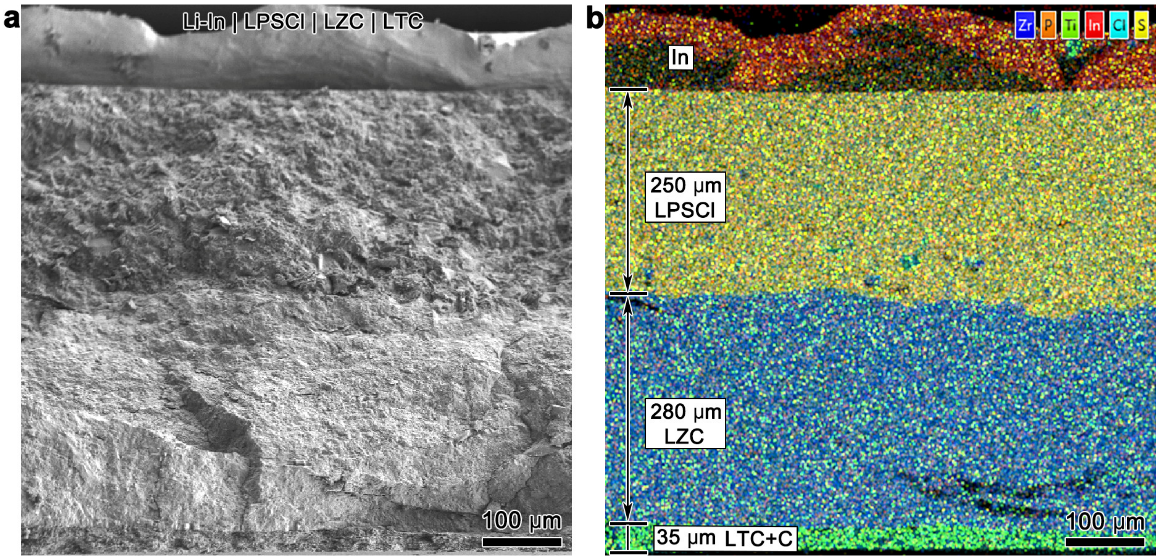

Supplementary Figure 14. a Secondary electron SEM image of the cross section of the Li-In | LPSCl | LZC | LTC cell. **b** The corresponding EDS mapping result.

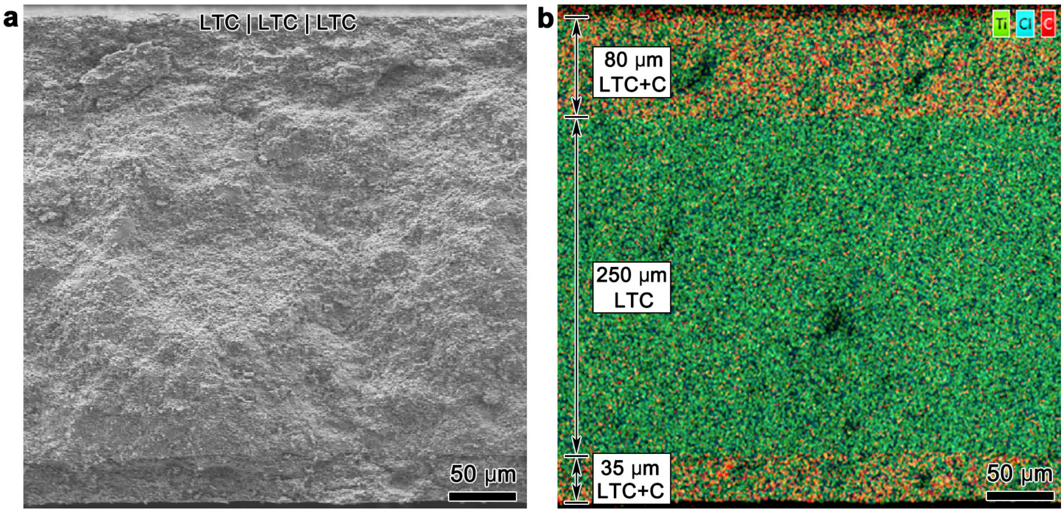

Supplementary Figure 15. a Secondary electron SEM image of the cross section of the single-LTC cell. **b** The corresponding EDS mapping result.

Supplementary Table 1. Rietveld refinement result from the XRD data of the as-milled LTC. The space group is *C2/m*. The refined lattice parameters are a = 6.350(11) Å, b = 10.882(2) Å, c = 6.337(10) Å, and β = 110.164(29)˚. The unit-cell volume is 411.00(12) Å^3.

Atoms	x	y	z	Occ.	site	Sym.	U_{iso} (Å^2)
Li1	0	0.170(5)	1/2	1	4*h*	2	0.211(10)
Li2	1/2	0	1/2	1	2*d*	2/m	0.211(10)
Ti1	0	0	0	0.851(8)	2*a*	2/m	0.037(6)
Ti2	0	0.333(8)	0	0.075(4)	4*g*	2	0.037(6)
Cl1	0.2410(12)	0.1615(6)	0.2328(9)	1	8*j*	1	0.0104(25)
Cl2	0.2573(17)	0	-0.2577(14)	1	4*i*	m	0.0104(25)

Supplementary Table 2. Rietveld refinement result from the XRD data of the 300 °C-annealed LTC. The space group is *C2/m*. The refined lattice parameters are *a* = 6.3499(16) Å, *b* = 10.8908(5) Å, *c* = 6.3526(15) Å, and β = 110.120(4)˚. The unit-cell volume is 412.504(11) $Å^3$.

Atoms	x	y	z	Occ.	site	Sym.	U_{iso} ($Å^2$)
Li1	0	0.170(4)	1/2	1	4*h*	2	0.108(7)
Li2	1/2	0	1/2	1	2*d*	2/m	0.108(7)
Ti1	0	0	0	0.754(4)	2*a*	2/m	0.0008(9)
Ti2	0	0.3295(22)	0	0.1230(21)	4*g*	2	0.0008(9)
Cl1	0.2421(5)	0.16265(28)	0.2364(4)	1	8*j*	1	0.0190(5)
Cl2	0.2406(7)	0	-0.2302(7)	1	4*i*	m	0.0190(5)

References

1. Sebti, E., Evans, H. A., Chen, H., Richardson, P. M., White, K. M., Giovine, R., Koirala, K. P., Xu, Y., Gonzalez-Correa, E., Wang, C., Brown, C. M., Cheetham, A. K., Canepa, P. & Clement, R. J. Stacking faults assist lithium-ion conduction in a halide-based superionic conductor. *J. Am. Chem. Soc.* **144**, 5795–5811 (2022).
2. Wu, E. A., Banerjee, S., Tang, H. M., Richardson, P. M., Doux, J. M., Qi, J., Zhu, Z. Y., Grenier, A., Li, Y. X., Zhao, E. Y., Deysher, G., Sebti, E., Nguyen, H., Stephens, R., Verbist, G., Chapman, K. W., Clément, R. J., Banerjee, A., Meng, Y. S. & Ong, S. P. A stable cathode-solid electrolyte composite for high-voltage, long-cycle-life solid-state sodium-ion batteries. *Nat. Commun.* **12**, 1256 (2021).
3. Lewis, J. A., Lee, C., Liu, Y., Han, S. Y., Prakash, D., Klein, E. J., Lee, H. W. & McDowell, M. T. Role of areal capacity in determining short circuiting of sulfide-based solid-state batteries. *ACS Appl. Mater. Inter.* **14**, 4051–4060 (2022).
4. Wang, Y., Hoang, B., Hoerauf, J., Lee, C., Lin, C. F., Rubloff, G. W., Lee, S. B. & Kozen, A. C. Hot and cold pressed LGPS solid electrolytes. *J. Electrochem. Soc.* **168**, 010533 (2021).
